# Supplementary material for: Clinical Significance of Lipoprotein Lipase (LPL) in People Living with HIV: A Comprehensive Assessment Including Lipidemia, Body Composition, Insulin Secretion, and Insulin Resistance
Source: Nutrients. 2025 Oct 13;17(20):3207. doi: 10.3390/nu17203207 (PMC12566720; doi:10.3390/nu17203207)
Supplement: Supplementary file 1 [file nutrients-17-03207-s001.zip › nutrients-3890096-supplementary.pdf]

Supplemental Table S1a. PLH categorized using an antilipemic drug or not (n = 48).

	All (n=48)		ALD (-) (n=33)		ALD (+) (n=15)			Statin (-) (n=37)		Statin (+) (n=11)			Fibrate (-) (n=42)		Fibrate (+) (n=6)		
	median/n	(IQR/%)	median/n	(IQR/%)	median/n	(IQR/%)	p	median/n	(IQR/%)	median/n	(IQR/%)	p	median/n	(IQR/%)	median/n	(IQR/%)	p
Age	44.0	(38.0-52.8)	43.0	(35.0-50.0)	52.0	(42.0-61.0)	**0.011** *	43.0	(35.5-51.5)	52.0	(42.0-61.0)	**0.025** *	43.5	(36.8-52.3)	52.0	(39.5-63.3)	0.180
LPL (ng/mL)	59.6	(46.0-73.3)	65.5	(46.6-79.5)	50.6	(42.4-59.3)	**0.024** *	62.8	(45.9-74.6)	50.6	(45.8-61.5)	0.173	62.5	(47.4-74.2)	43.4	(35.4-56.1)	**0.038** *
GPIHBP1(pg/mL)	906.0	(726.9-1094.8)	951.0	(735.3-1092.5)	828.5	(671.1-1169.3)	0.519	935.8	(735.3-1092.5)	781.5	(647.5-1198.0)	0.486	920.9	(728.1-1095.9)	845.1	(637.8-1024.1)	0.492
HTGL (ng/mL)	45.8	(33.7-61.0)	41.1	(30.4-57.4)	51.4	(45.1-63.8)	**0.039** *	42.1	(31.1-57.4)	56.0	(45.1-76.7)	0.053	43.6	(31.9-64.0)	50.9	(45.2-60.2)	0.408
IRI (μU/mL)	7.5	(3.8-9.2)	5.8	(3.7-9.1)	8.1	(4.9-11.3)	0.127	6.6	(3.8-8.7)	8.8	(4.0-12.9)	0.286	5.9	(3.7-9.6)	8.4	(8.0-14.0)	0.073
FRTN (ng/mL)	88.3	(58.8-181.1)	90.5	(59.0-146.0)	86.1	(52.6-213.2)	0.859	90.8	(59.0-192.3)	71.6	(52.6-151.9)	0.425	85.3	(58.9-145.6)	207.6	(48.0-228.9)	0.436
FPG (mg/dL)	96.0	(90.3-100.8)	94.0	(89.0-99.0)	99.0	(95.0-117.0)	**0.020** *	95.0	(89.5-99.0)	99.0	(96.0-110.0)	**0.037** *	96.0	(90.0-99.3)	101.0	(93.8-117.3)	0.249
HOMA-IR	1.7	(0.9-2.3)	1.4	(0.9-2.0)	2.1	(1.2-3.1)	0.074	1.6	(0.9-2.2)	2.1	(0.9-3.7)	0.243	1.4	(0.9-2.2)	2.3	(2.0-3.5)	**0.042** *
HOMA-β	65.3	(48.4-103.9)	64.2	(39.3-107.7)	72.0	(49.4-96.8)	0.929	64.2	(44.5-106.0)	72.0	(48.0-96.0)	0.883	63.2	(44.7-101.3)	86.6	(53.6-157.1)	0.249
Diabetes	4	(8.3)	1	(3.0)	3	(20.0)	**0.049** *	2	(5.4)	2	(18.2)	0.178	2	(4.8)	2	(33.3)	**0.018** *
Hypertension	12	(25.0)	6	(18.2)	6	(4.0)	0.106	8	(21.6)	4	(36.4)	0.322	10	(23.8)	2	833.3)	0.614
TG (mg/dL)	107.0	(74.8-155.5)	104.0	(66.0-147.5)	129.0	(100.0-236.0)	0.083	107.0	(71.0-159.5)	107.0	(93.0-134.0)	0.825	105.0	(71.5-144.3)	178.5	(127.3-269.5)	**0.016** *
TG≧150 (mg/dL)	13	(27.1)	8	(24.2)	5	(27.1)	0.511	11	(29.7)	2	(18.2)	0.449	10	(23.8)	3	(50.0)	0.177
HDL-C (mg/dL)	51.5	(42.8-60.0)	50.0	(42.0-62.0)	53.0	(42.5-58.0)	0.851	49	(41.3-60.0)	53	(43.0-59.5)	0.873	53	(44.0-62.0)	43	(41.5-51.5)	0.221
LDL-C (mg/dL)	117.5	(100.3-135.8)	115.0	(97.0-136.0)	119.0	(104.0-131.0)	0.640	117	(97.0-137.5)	119	(104.0-128.0)	0.980	115	(99.0-135.3)	120.5	(109.3-147.5)	0.418
Antilipemic drug	15	(31.3)	0	(0.0)	15	(100.0)	NA	4	(10.8)	11	(100.0)	**<0.001** *	9	(21.4)	6	(100.0)	**<0.001** *
Statin	11	(22.9)	0	(0.0)	11	(73.3)	NA	0	(0.0)	11	(100.0)	NA	9	(21.4)	2	(33.3)	0.516
Fibrate	5	(10.4)	0	(0.0)	5	(33.3)	NA	4	(10.8)	2	(18.2)	0.516	0	(0.0)	6	(100.0)	NA
Ht (cm)	172.0	(168.0-176.0)	173.0	(168.0-177.0)	171.0	(162.0-174.0)	0.095	173	(168.0-177.0)	171	(168.0-173.0)	0.188	172.5	(168.0-176.3)	170	(161.0-174.8)	0.305

BW (kg)	71.0	(63.9-79.0)	70.0	(62.4-81.8)	73.3	(66.2-76.9)	0.772	70	(62.4-81.0)	73.3	(66.2-76.9)	0.768	71	(63.7-78.3)	71.45	(64.8-82.5)	0.796
BMI (kg/m²)	24.4	(21.4-27.0)	24.0	(20.9-27.0)	24.8	(23.5-27.0)	0.247	24	(21.1-27.0)	24.8	(23.5-27.0)	0.371	24.2	(21.2-26.9)	25.45	(23.4-27.6)	0.418
BMI≧25 (kg/m²)	21	(43.8)	14	(42.4)	7	(46.7)	0.784	16	(43.2)	5	(45.5)	0.897	18	(42.9)	3	(50.0)	0.741
SMI (kg/m²)	8.1	(7.3-8.6)	7.9	(7.3-8.5)	8.2	(7.4-8.6)	0.755	7.9	(7.3-8.5)	8.2	(7.4-8.6)	0.572	7.95	(7.3-8.4)	8.4	(7.3-8.7)	0.512
FMI (kg/m²)	6.4	(4.2-8.3)	5.9	(3.9-8.4)	6.9	(6.0-7.9)	0.281	6.3	(4.1-8.4)	6.9	(5.5-8.2)	0.548	6.1	(4.2-8.4)	7.3	(5.9-7.8)	0.659
SMI/FMI	1.3	(1.0-1.8)	1.4	(1.0-2.0)	1.2	(1.0-1.3)	0.265	1.3	(1.0-1.9)	1.2	(1.0-1.4)	0.640	1.3	(1.0-1.8)	1.05	(1.0-1.6)	0.659
Body fat (%)	25.1	(19.1-29.9)	22.8	(18.0-30.1)	26.4	(24.9-30.0)	0.296	23.1	(18.3-30.3)	26.4	(24.9-28.5)	0.508	24.95	(18.8-29.8)	26.35	(18.4-30.3)	0.915
WC (cm)	84.7	(80.8-93.9)	82.5	(79.2-94.6)	88.2	(84.0-94.3)	0.213	83.2	(79.8-93.6)	88.2	(84.0-95.0)	0.339	84.45	(80.5-93.4)	88.5	(81.5-94.9)	0.064
CD4	580.5	(466.3-730.8)	599.0	(460.5-720.0)	547.0	(482.0-844.0)	0.798	599	(460.5-720.0)	547	(482.0-894.0)	0.704	600	(460.8-752.5)	523.5	(463.5-722.8)	0.055
HIV-RNA<20 (c/ml)	45	(93.7)	30	(90.9)	15	(100.0)	0.427	34	(91.9)	0	(0.0)	0.601	39	(92.9)	0	(100.0)	0.244
BIC	19	(39.6)	15	(45.5)	4	(26.7)	0.217	15	(40.5)	4	(36.4)	0.804	18	(42.9)	1	(16.7)	0.220
DTG	16	(33.3)	8	(24.2)	8	(53.3)	**0.048** *	10	(27.0)	6	(54.5)	0.089	13	(31.0)	3	(50.0)	0.355
EVG/c	2	(4.2)	0	(0.0)	2	(13.3)	**0.032** *	1	(2.7)	1	(9.1)	0.352	1	(2.4)	1	(16.7)	0.101
RAL	3	(6.3)	3	(9.1)	0	(0.0)	0.228	3	(8.1)	0	(0.0)	0.329	3	(7.1)	0	(0.0)	0.499
INSTI	40	(83.3)	26	(78.8)	14	(93.3)	0.210	29	(78.4)	11	(100.0)	0.091	35	(83.3)	5	(83.3)	1.000
DRV	8	(16.7)	5	(15.2)	3	(20.0)	0.676	6	(16.2)	2	(18.2)	0.878	7	(16.7)	1	(16.7)	1.000
TAF/FTC	33	(68.8)	24	(72.7)	9	(60.0)	0.378	26	(70.3)	7	(63.6)	0.677	29	(69.0)	4	(66.7)	0.906
3TC	12	(25.0)	7	(21.1)	5	(33.3)	0.369	9	(24.3)	3	(27.3)	0.843	10	(23.8)	2	(33.3)	0.614

Statistically significant differences were indicated with a $p < 0.05$ value and marked with a bold red and *. Abbreviations: SMI—skeletal muscle mass index; FMI—fat mass index; CD4—counts of CD4-positive T-lymphocytes; BIC—bictegravir; DTG—dolutegravir; EVG/c—elvitegravir combined with cobicistat; RAL—raltegravir; INSTIs—integrase strand transfer inhibitors; DRV—darunavir; TAF/FTC—emtricitabine/tenofovir alafenamide fumarate; 3TC—lamivudine.

Supplemental Table S1b. PLH categorized by insulin secretion, resistance, and BMI (n = 48).

	HOMA-β<80(n=30)		HOMA-β≧80(n=18)			HOMA-IR<2.5(n=37)		HOMA-IR≧2.5(n=11)			BMI<25(n=27)		BMI≧25(n=21)		
	median/n	(IQR/%)	median/n	(IQR/%)	p	median/n	(IQR/%)	median/n	(IQR/%)	p	median/n	(IQR/%)	median/n	(IQR/%)	p
Age	48.0	(37.8-55.3)	42.5	(37.5-50.3)	0.241	44.0	(38.0-52.5)	46.0	(36.0-56.0)	0.844	43	(37.0-52.0)	46.0	(40.0-54.5)	0.466
LPL (ng/mL)	62.3	(50.3-73.3)	47.6	(39.8-73.4)	0.084	61.7	(47.4-73.3)	50.3	(41.7-77.8)	0.338	62.5	(48.3-73.6)	50.3	(45.4-81.0)	0.496
GPIHBP1 (pg/mL)	906.0	(726.9-1092.3)	881.7	(717.8-1097.1)	0.800	852.5	(726.2-1064.9)	1093.7	(735.8-1153.6)	0.298	901.5	(706.7-1094.0)	950.7	(751.0-1146.8)	0.384
HTGL (ng/mL)	43.2	(33.7-61.8)	48.4	(31.3-62.2)	0.936	43.6	(31.9-60.2)	49.7	(41.7-71.1)	0.298	41.6	(31.6-61.8)	49.7	(40.6-73.5)	0.220
IRI (μU/mL)	4.1	(3.0-6.8)	10.9	(8.6-19.4)	<0.001 *	5.6	(3.6-7.8)	15.1	(12.9-29.1)	<0.001 *	4.2	(2.5-9.5)	9.3	(6.2-14.3)	<0.001 *
FRTN (ng/mL)	84.1	(52.6-120.3)	122.6	(62.4-259.2)	0.053	82.2	(55.1-146.0)	100.0	(64.9-343.5)	0.138	90.5	(56.0-181.3)	86.1	(59.1-209.5)	0.561
FPG (mg/dL)	96.0	(90.8-101.0)	96.5	(90.0-101.8)	0.848	94.0	(89.5-99.0)	99.0	(97.0-122.0)	0.003 *	94	(89.0-100.0)	99.0	(92.0-105.5)	0.058
HOMA-IR	0.9	(0.8-1.7)	2.7	(2.0-4.8)	<0.001 *	1.4	(0.9-1.9)	4.0	(3.1-11.8)	<0.001 *	0.9	(0.6-2.7)	2.3	(1.5-3.9)	<0.001 *
HOMA-β	51.0	(38.7-62.7)	121.1	(99.2-164.4)	<0.001 *	56.0	(39.3-75.2)	151.0	(99.3-252.0)	<0.001 *	52.4	(37.5-103.3)	99.3	(55.0-142.5)	0.002 *
Diabetes	3	(10.0)	1	(5.6)	0.590	2.0	(5.4)	2.0	(18.2)	0.178	2	(7.4)	2	(9.5)	0.792
Hypertension	7	(23.3)	5	(27.8)	0.731	7.0	(18.9)	5.0	(45.5)	0.074	4	(14.8)	8	(38.1)	0.065
TG (mg/dL)	98.0	(68.0-137.8)	145.0	(101.3-255.3)	0.012 *	106.0	(75.5-138.5)	163.0	(72.0-254.0)	0.048 *	96.0	(70.0-155.0)	140.0	(99.5-236.0)	0.013 *
TG≧150(mg/dL)	4	(13.3)	9	(50.0)	0.006 *	6.0	(16.2)	7.0	(63.6)	0.002 *	3	(11.1)	10	(47.6)	0.005 *
HDL-C (mg/dL)	56.0	(45.0-64.0)	46.0	(41.0-50.0)	0.060	55.0	(43.0-64.5)	48.0	(36.5-51.5)	0.070	56.0	(45.0-60.5)	46.0	(41.0-56.0)	0.108
LDL-C (mg/dL)	120.5	(92.8-136.8)	112.0	(104.8-125.0)	0.632	118.0	(97.0-137.5)	112.0	(104.0-124.0)	0.624	112.0	(90.0-135.5)	124.0	(111.5-137.5)	0.020 *
Antilipemic drug	9	(30.0)	6.000	(33.3)	0.809	11.0	(29.7)	4.0	(36.4)	0.677	8	(29.6)	7	(33.3)	0.784
Statin	7	(23.3)	4	(22.2)	0.929	7.0	(18.9)	4.0	(36.4)	0.227	6	(22.2)	5	(23.8)	0.897
Fibrate	3	(10.0)	2	(11.1)	0.903	5.0	(13.5)	0.0	(0.0)	0.198	3	(11.1)	2	(9.5)	0.858
Ht (cm)	172.0	(168.0-175.3)	173.0	(166.0-177.8)	0.798	172.0	(168.0-175.0)	175.0	(169.0-180.0)	0.253	172.0	(168.0-176.0)	172.0	(167.5-177.0)	0.787
BW (kg)	68.0	(63.7-73.5)	79.6	(66.4-90.0)	0.021 *	68.2	(63.1-74.3)	84.6	(76.9-95.2)	<0.001 *	65.0	(60.3-79.0)	80.0	(73.0-88.1)	<0.001 *

BMI (kg/m²)	23.7	(21.0-25.3)	26.9	(24.5-29.3)	**0.002**	*	23.8	(20.9-25.5)	27.3	(26.6-29.3)	**<0.001**	*	21.6	(20.7-27.4)	27.3	(26.3-29.2)	**<0.001**	*
BMI≧25(kg/m²)	8	(26.7)	13	(72.2)	**0.002**	*	11.0	(29.7)	10.0	(90.9)	**<0.001**	*	0	(0.0)	21	(100.0)	NA	
SMI (kg/m²)	7.9	(7.3-8.2)	8.4	(7.4-8.8)	0.060		7.8	(7.3-8.2)	8.6	(8.2-9.0)	**0.001**	*	7.5	(7.1-8.1)	8.5	(8.2-8.9)	**<0.001**	*
FMI (kg/m²)	5.6	(3.9-7.4)	7.7	(6.7-10.4)	**0.003**	*	5.9	(3.9-7.6)	8.2	(6.9-10.9)	**0.004**	*	4.8	(3.8-8.4)	8.2	(6.8-9.7)	**<0.001**	*
SMI/FMI	1.4	(1.1-1.9)	1.1	(0.9-1.3)	**0.007**	*	1.4	(1.0-2.0)	1.1	(0.9-1.2)	**0.019**	*	1.6	(1.1-1.3)	1.0	(0.9-1.3)	**<0.001**	*
Body fat (%)	21.7	(18.0-26.8)	27.7	(25.6-33.4)	**0.002**	*	22.8	(18.0-28.1)	28.1	(26.4-34.1)	**0.003**	*	20.7	(17.2-29.1)	28.1	(25.2-32.3)	**<0.001**	*
WC (cm)	83.2	(79.3-88.3)	93.4	(83.5-100.0)	**0.003**	*	83.2	(79.2-90.1)	96.5	(91.0-109.1)	**<0.001**	*	81.9	(76.5-93.7)	92.8	(89.0-100.3)	**<0.001**	*
CD4	620.0	(492.3-826.0)	517.0	(457.0-694.8)	0.198		639.0	(481.5-814.5)	508.0	(461.0-673.0)	0.215		599.0	(427.0-730.5)	562.0	(493.5-714.0)	0.843	
HIV-RNA<20 (c/ml)	29	(96.7)	16	(88.9)	**0.011**	*	35.0	(94.6)	10.0	(90.9)	0.249		25	(92.6)	20	(95.2)	0.121	
BIC	13	(43.3)	6	(33.3)	0.493		15.0	(40.5)	4.0	(36.4)	0.804		11	(40.7)	8	(38.1)	0.853	
DTG	10	(33.3)	6	(33.3)	1.000		11.0	(29.7	5.0	(45.5)	0.331		8	(29.6)	8	(38.1)	0.537	
EVG/c	1	(3.3)	1	(5.6)	0.709		2.0	(5.4)	0.0	(0.0)	0.431		1	(3.7)	1	(4.8)	0.856	
RAL	2	(6.7)	1	(5.6)	0.878		2.0	(5.4)	1.0	(9.1)	0.658		1	(3.7)	2	(9.5)	0.409	
INSTI	26	(86.7)	14	(77.8)	0.424		30.0	(81.1)	10.0	(90.9)	0.443		21	(77.7)	19	(90.5)	0.242	
DRV	5	(16.7)	3	(16.7)	1.000		7.0	(18.9)	1.0	(9.1)	0.443		6	(22.2)	2	(9.5)	0.242	
TAF/FTC	22	(73.3)	11	(61.1)	0.376		27.0	(73.0)	6.0	(54.5)	0.247		20	(74.1)	13	(61.9)	0.367	
3TC	7	(23.3)	5	(27.8)	0.731		8.0	(21.6)	4.0	(36.4)	0.322		5	(18.5)	7	(33.3)	0.240	

Statistically significant differences were indicated with a $p < 0.05$ value and marked with a bold red and *. Abbreviations: SMI—skeletal muscle mass index; FMI—fat mass index; CD4—counts of CD4-positive T-lymphocytes; BIC—bictegravir; DTG—dolutegravir; EVG/c—elvitegravir combined with cobicistat; RAL—raltegravir; INSTIs—integrase strand transfer inhibitors; DRV—darunavir; TAF/FTC—emtricitabine/tenofovir alafenamide fumarate; 3TC—lamivudine.

Supplemental Table S2. Reconstructed Table 3 with Bonferroni-Adjusted p-values.

This table reproduces the SPSS 'Variables in the Equation' output, incorporating Bonferroni-adjusted p-values as calculated from the provided data. Since all adjusted p-values exceed the corrected threshold (α_adj = 0.05/number of variates in each analysis).

**For ALD (+)**	B	standard error	Wald	degree of freedom	Intentional probability	Exp(B) =aOR	95% CI for EXP(B) =Lower to upper limit of aOR		Adjusted p-value
Age	0.153	0.059	6.676	1	0.01	1.165	1.038	1.309	0.06
LPL (ng/mL)	-0.09	0.037	6.021	1	0.014	0.914	0.85	0.982	0.084
HTGL (ng/mL)	0.002	0.017	0.009	1	0.923	1.002	0.969	1.035	1
FBS	0.007	0.031	0.047	1	0.829	1.007	0.947	1.071	1
Diabetes	-0.23	1.877	0.015	1	0.903	0.795	0.02	31.487	1
DTGorEVG	-0.665	0.994	0.448	1	0.503	0.514	0.073	3.609	1
**For Statin (+)**									
Age	0.074	0.041	3.297	1	0.069	1.077	0.994	1.167	0.138
FPG	0.018	0.02	0.803	1	0.37	1.018	0.979	1.059	0.74
**For Fibrate (+)**									
LPL(ng/mL)	-0.05	0.054	0.848	1	0.357	0.952	0.856	1.057	1
HOMA-IR	-2.792	1.351	4.272	1	0.039	0.061	0.004	0.866	0.156
Diabetes	8.326	3.707	5.045	1	0.025	4128.096	2.888	5900350.401	0.1
TG	0.043	0.023	3.517	1	0.061	1.043	0.998	1.091	0.244
**For BMI≧25**									
IRI(μU/mL)	0.162	0.069	5.602	1	0.018	1.176	1.028	1.345	0.0540
TG	0.006	0.005	1.142	1	0.285	1.006	0.995	1.016	0.855
LDL	0.055	0.021	6.611	1	0.01	1.057	1.013	1.102	0.03
**For HOMA-β≧80**									

TG	0.014	0.007	4.686	1	0.03	1.014	1.001	1.027	0.24
BW(kg)	0.116	0.148	0.612	1	0.434	1.123	0.84	1.5	1
BMI	0.107	0.353	0.091	1	0.763	1.112	0.557	2.224	1
FMI	0.597	0.518	1.328	1	0.249	1.817	0.658	5.017	1
Body Fat Percentage	0.15	0.158	0.897	1	0.344	1.162	0.852	1.584	1
Waist circumference (cm)	-0.203	0.216	0.877	1	0.349	0.817	0.534	1.248	1
SMI1perFMI	1.737	1.664	1.09	1	0.297	5.679	0.218	148.034	1
HIVRNAbelow20	-3.3	1.804	3.346	1	0.067	0.037	0.001	1.266	0.536
**For HOMA-IR ≧ 2.5**									
TG	0.012	0.009	1.671	1	0.196	1.012	0.994	1.031	1
BW(kg)	0.099	0.248	0.158	1	0.691	1.104	0.678	1.796	1
BMI	3.703	2.094	3.126	1	0.077	40.564	0.669	2459.779	0.616
SMI (InBody automatic calculation)	-4.338	4.467	0.943	1	0.331	0.013	0	82.866	1
FMI	-4.033	2.523	2.554	1	0.11	0.018	0	2.491	0.88
SMI1perFMI	-1.63	6.02	0.073	1	0.787	0.196	0	26078.052	1
Body Fat Percentage	0.736	0.487	2.284	1	0.131	2.087	0.804	5.421	1
Waist circumference (cm)	-0.326	0.299	1.19	1	0.275	0.722	0.402	1.297	1

Interpretation and Reporting Suggestion

After Bonferroni correction, none of the variables remained statistically significant.
